# Trends in country and gender representation on editorial boards in anaesthesia journals: a pooled cross‐sectional analysis

**DOI:** 10.1111/anae.15733

**Published:** 2022-05-05

**Authors:** Anaesthesia Journal Editorial Board Diversity and Representation Study Group, M. D. Bould, R. Eng, S. J. Glaze, S. Kudsk‐Iversen, S. McLure, O. Higgins‐Gill, R. Inglis, S. Krouch, N. Shamambo, V. Thwaites

**Keywords:** diversity, editorial board, gender, LMIC, representation

## Abstract

Evidence exists that women and people from low‐ and middle‐income countries are under‐represented on the editorial boards of medical journals. This may adversely influence the journal output. We conducted a pooled, cross‐sectional evaluation of the editorial board membership of anaesthesia journals. We collected data on members of editorial boards from the founding year and at 5‐yearly intervals until 2020. For each editor, we recorded gender, country of affiliation, World Bank income classification (1990 onwards) and editorial role (2020 only). The composite editorial board diversity score was calculated for each editorial board. We obtained complete data for the composition of editorial boards from all 30 journals for 2020, but for only 171 out of 304 editorial boards (56%) over the time period examined. In 2020, 409 out of 1973 (21%) were women (range across the editorial boards 0–39%) and 139 out of 1982 (7%) were from low‐, low‐middle‐ and upper‐middle‐income countries (range across the editorial boards 0–71%). In 2020, of editorial board positions with known seniority status, 109 out of 259 (42%) of women and 306 out of 960 (32%) of men were in senior roles. In the same year, 397 out of 1115 (36%) of people from high‐income countries were in senior roles, compared with 19 out of 93 (20%) of people from upper‐middle‐income countries and 0 out of 14 (0%) people from lower‐middle‐income countries. The median composite editorial board diversity score was 4 (range 2–6) in 2020 – 5 or less suggests poor diversity, while 8 or more suggests good diversity. Women and people from low‐ and middle‐income countries are under‐represented on anaesthesia journal editorial boards. The editorial boards do not reflect the anaesthesia workforce and may act as a barrier to the publication of research produced by these groups. Urgent action is required to improve diversity.

## Introduction

Editorial boards are the gatekeepers to research output. Journal editors are described as a “*team of experts in their field*” [1], and their recruitment is generally via existing members of the editorial board. They shape journal policy and strategy. The decision regarding which manuscripts to accept for publication lies with the judgement of editorial boards in the peer review process [2]. Publications provide a metric for advancement in academic medicine and rates of acceptance influence career progress [3]. A lack of diversity in editorial boards is likely to impair the research careers of individuals with particular characteristics, undervaluing their perspectives and contributions. This not only affects those individual researchers and their advancement, but determines which knowledge is prioritised, leading to further marginalisation of under‐represented groups [4]. Evidence already exists that women and people from institutions in low‐ and middle‐income countries are under‐represented in the editorial boards of major journals [5, 6, 7]. This is also true of authorship in journals [8, 9, 10] and, specific to anaesthesia, it has been shown that women are under‐represented as editors of journals [11]. This lack of diversity may translate to a loss of experience and knowledge relevant to publications.

In a recent consensus statement about measures to promote equitable authorship in the publication of research from international partnerships, Morton et al. highlight the problem of ‘parachute’ research, which is “*the practice of conducting primary research within a host country and subsequently publishing findings with inadequate recognition of local researchers, staff and/or supporting infrastructure*” [12]. The authors identify the lack of diversity in editorial boards as one of many issues perpetuating this practice. Since members of editorial boards are often established researchers [3], a lower frequency of publication may hinder authors who are women and/or from low‐ or middle‐income country institutions in advancing to editorial board positions. A cycle therefore exists: these under‐represented groups are less likely to have had work published, less likely to advance to editorial board positions and so cannot influence future authorship, publications and editorial board membership [3, 13].

We aimed to explore the diversity of the editorial boards of major anaesthesia journals. We considered the gender balance and geographical distribution of institutional affiliation of editorial board members as well as the representation in senior positions. We also calculated a composite editorial board diversity score [6] for each editorial board to facilitate comparison between journals. Where possible, historical data were obtained to assess for trends.

## Methods

We used pooled cross‐sectional analysis to evaluate the editorial board membership of journals in the field of anaesthesia. The study received an exemption from ethical review. A reflexivity statement on equitable research partnerships as outlined by Morton et al. [12] is available in online Supporting Information (Appendix S1).

We evaluated editorial board members based on gender, country of affiliation and seniority. Additionally, where data were available, we calculated the composite editorial board diversity score, based on previously described metrics [6]. We included all journals under the category ‘Anaesthesiology’ from the InCites Journal Citation Reports (available via Clarivate™, London, UK) in 2018. At this time, only the Science Citation Index Expanded was included in this listing. For each journal, we aimed to include data from the year the journal was first printed up to the year 2020 in 5‐year intervals. Data collection started in September 2020 and was completed in June 2021. We omitted journal names in the final data, as we did not want this to impair response rates to queries.

For current (2020) editorial boards, we used the journal website. For other years, we obtained the data using the following steps: a review of editorial board as published in the journal, either online or print issues (accessed via the University of Oxford) or if the editorial board was not listed online or available in print, we contacted the journal editorial office. If no reply, a minimum of two further attempts were made to contact the journal before deeming those data ‘unavailable’.

The country of affiliation of the editorial board member's institution was categorised according to World Bank geographical regions and World Bank Income classification group in the year of publication to determine whether it is a high‐income country (HIC), upper‐middle‐income country (UMIC), lower‐middle‐income country (LMIC), or low‐income country (LIC) [14]. This classification began in 1986, so has been applied for data collected from 1990 onwards [15]. We collected the data using the following steps: as reported in the editorial board listing, by institution; citation search and affiliation as listed on the most recent publication of the year of question; and any website listing the editor in an academic role alongside their institution. If this did not yield an answer, the country of affiliation was documented as ‘unknown’.

We attempted to categorise gender in a simplified binary approach, which is an approximation given the constraints of the available information. *“Gender refers to the characteristics of women, men, girls and boys that are socially constructed. This includes norms, behaviours and roles associated with being a woman, man, girl or boy. Gender interacts with but is different from sex, which refers to the different biological and physiological characteristics of females, males and intersex persons, such as chromosomes, hormones and reproductive organs*.”[16]. Gender was derived from one or more of the following methods (based on previously described approach [17]): assessment of the given names of the editor for typical gender association; online search to find editor's requested use of gender‐specific pronouns or other references to their gender in text or photographs; or online search of the editor's given name for typical gender assignment using genderchecker.com.

We acknowledge that this approach makes flawed assumptions based on typical gender associations of names or appearances. It also assumes a binary gender rather than accommodating the true range of possibilities.

We collected data on the roles assigned to each member of the editorial board. Due to the wide variety of roles listed [18], we contacted the journal editorial offices to clarify which roles were senior and whether they were included in the editorial board. If no reply was received, a further two attempts at contact were made before deeming the seniority of a role as ‘unknown’.

Following data collection for editorial boards and their members, we calculated the composite editorial board diversity score. This was first proposed by Bhaumik and Jagnoor [6] for use in global health journals and scores the diversity of an editorial board in the domains of gender (up to 4 points), country income level (up to 3 points) and geographic region (up to 3 points). Higher scores are awarded for greater diversity, with an overall maximum score of 10 for a given editorial board. If the composite editorial board diversity score is ≤5, it indicates poor diversity of the editorial board, 6 or 7 is moderate and 8 or more is good diversity.

International collaboration on data collection was enabled with Google Drive (Google LLC, Menlo Park, CA, USA). Data handling was performed using Microsoft Excel (version 2016, Microsoft Corporation, Redmond, WA, USA). All analyses were purely descriptive and no statistical hypotheses formulated or tested.

## Results

**Figure 1:**
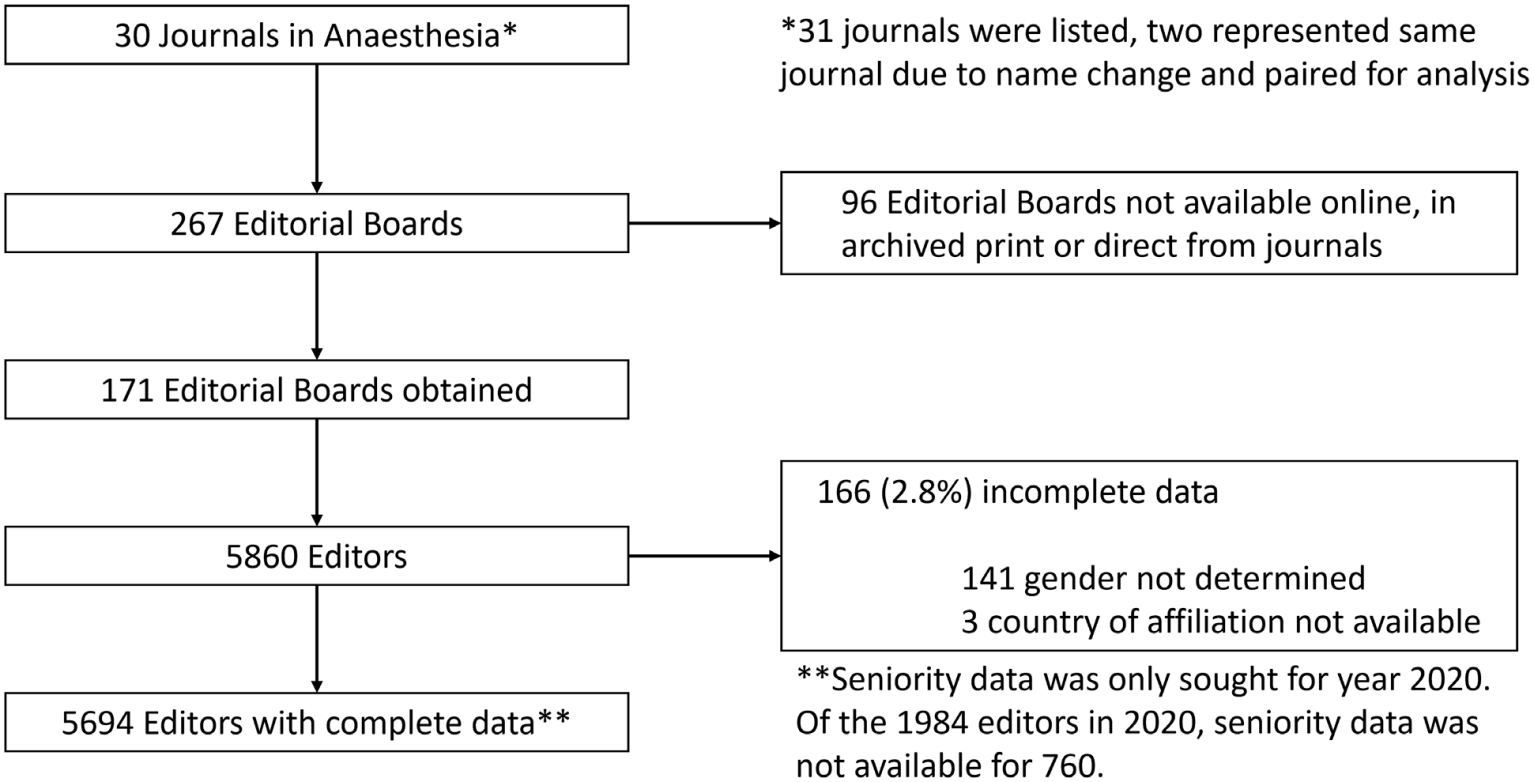
Flow chart of data inclusion and exclusion.

**Figure 2:**
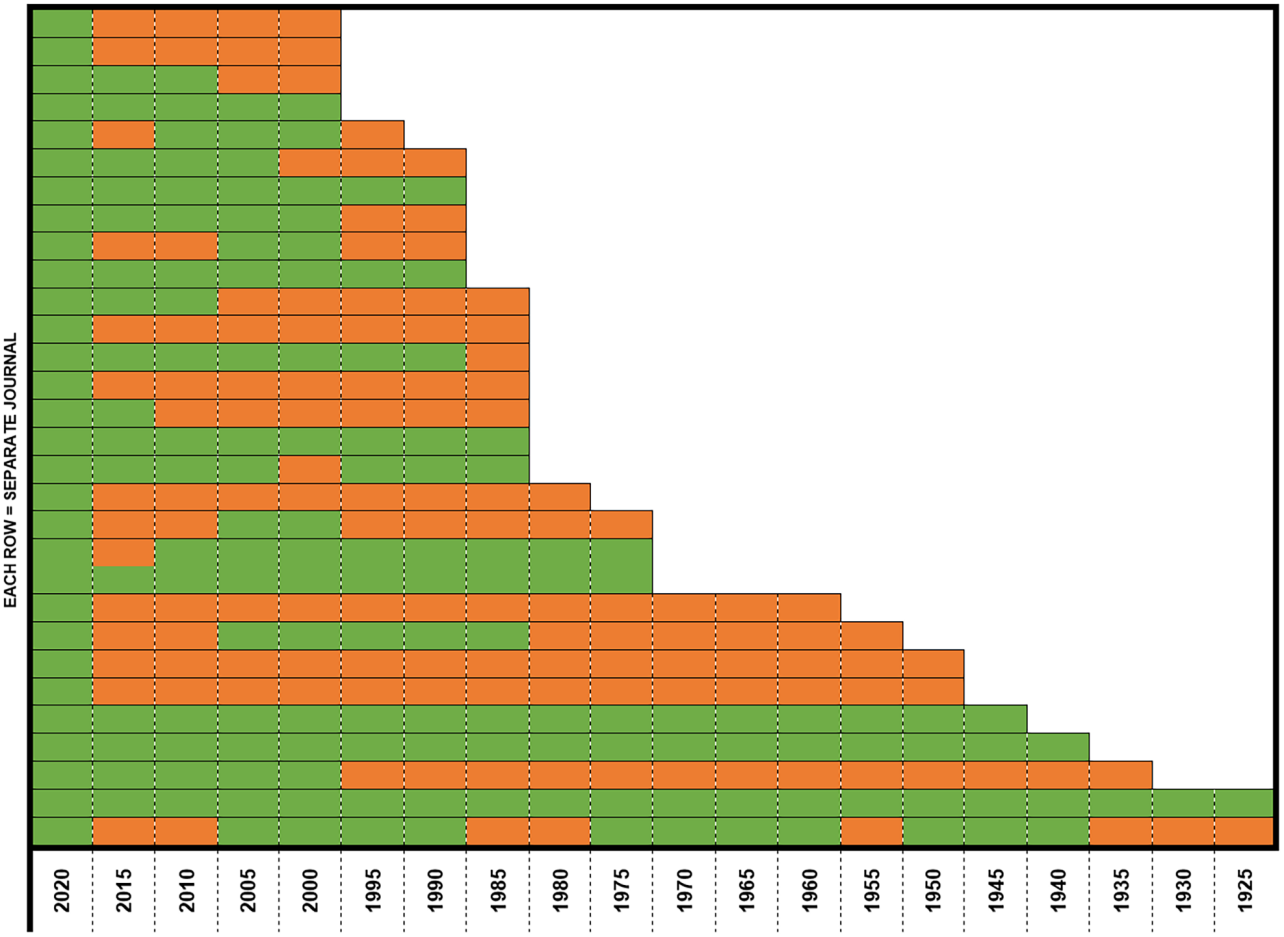
Availability of data for current and historical editorial boards for each included journal since they started. Green, editorial board data available; red, editorial board data not available. [Colour figure can be viewed at wileyonlinelibrary.com]

A total of 30 journals in anaesthesia were included (Fig. 1). Out of a possible 304 editorial boards from all years assessed, we were able to obtain data from 171 (56%) from years ranging 1925 to 2020 (Fig. 2), including all the data for 2020. The median (IQR [range]) editorial board size was 23 (10–45 [1–171]). Out of 5860 editors, we could not determine gender for 141, country of affiliation for 3 and both gender and country of affiliation for 22.

Across the data, a total of 131 different editorial roles were listed, with ‘editorial board’ (n = 1578), ‘editor’ (n = 960), ‘associate editor’ (n = 947), ‘editorial advisory board’ (n = 569) and ‘section editor’ (n = 388) the most common. Out of the 131 roles, 101 were used less than 10 times. We contacted all the journals for clarification of which roles used in 2020 were senior, not senior or not formally part of the editorial board. Out of the 21 who replied, 19 clarified all roles and two clarified some roles.

**Figure 3:**
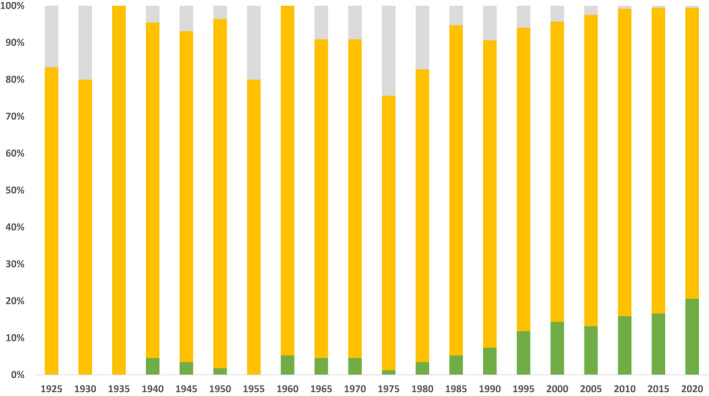
Proportion of editors of different gender across the available data. Grey, data not available; yellow, men; green, women. [Colour figure can be viewed at wileyonlinelibrary.com]

Based on available data, the proportion of women across all anaesthesia journal editorial boards has gradually increased over the years to 21% in 2020 (Fig. 3). In 2020, their representation on individual editorial boards ranged from 0 to 39% (see online Supporting Information, Table S1).

**Figure 4:**
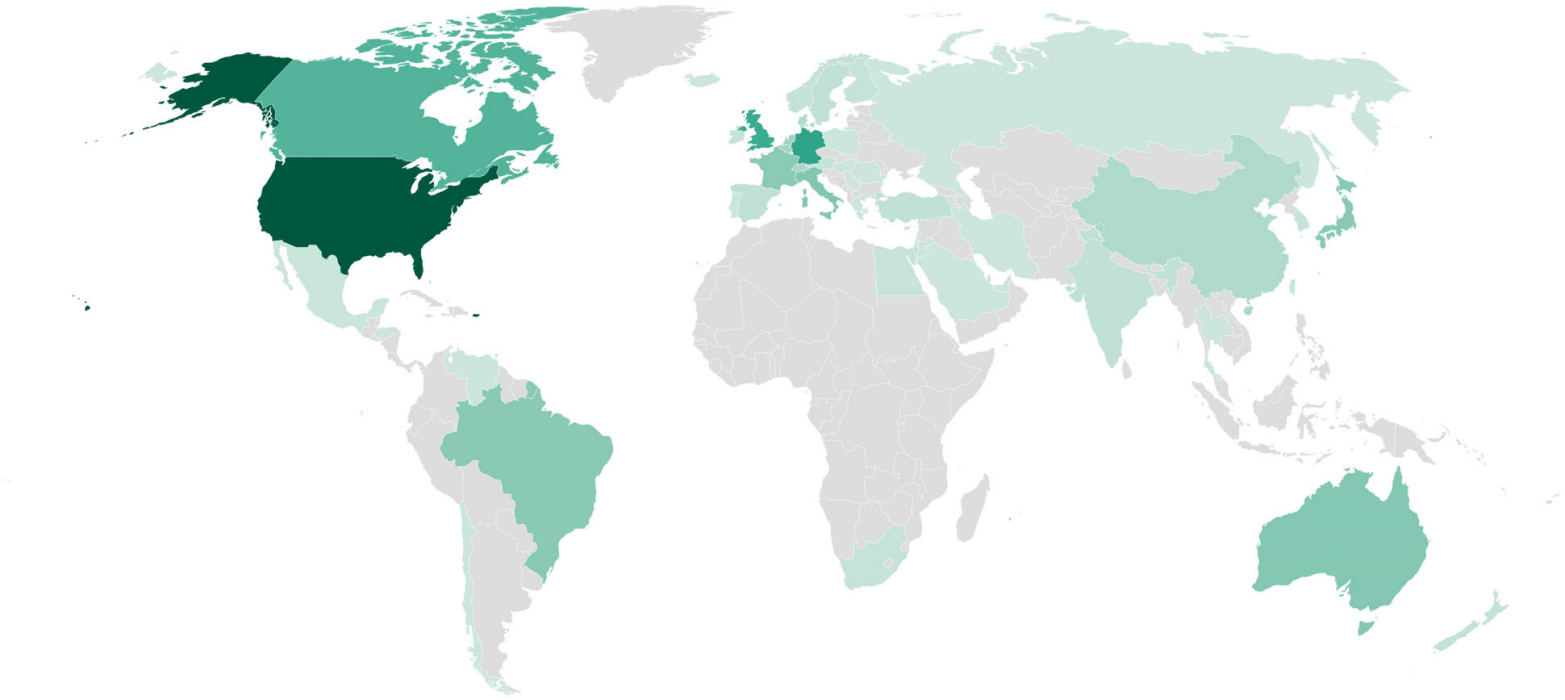
Choropleth map of the world, coloured according to the number of editors affiliated with each country in 2020. Map created with Datawrapper (Datawrapper GmbH, Germany). [Colour figure can be viewed at wileyonlinelibrary.com]

Over 60% of editorial board members were affiliated with institutions from the USA or the UK (3529 out of 5860, see online Supporting Information, Table S2). This number decreased to 53% when looking at 2020 alone (1063 out of 1984) (Fig. 4).

**Figure 5:**
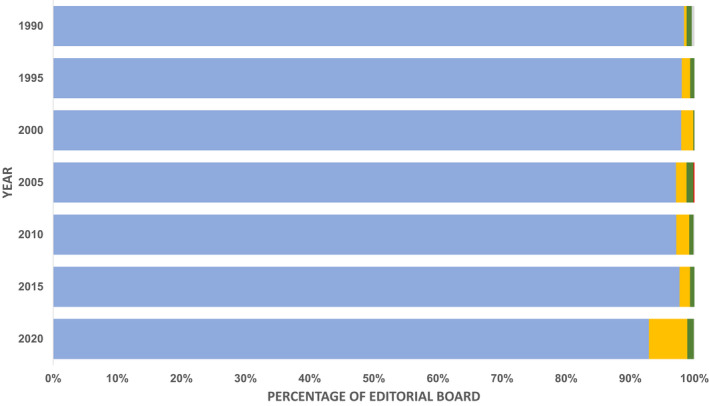
Proportion of editors affiliated with countries in different World Bank income classifications in the years where data are available. Blue, high‐income countries; yellow, upper‐middle‐income countries; green, lower‐middle‐income countries; red, low‐income countries; grey, data not available. [Colour figure can be viewed at wileyonlinelibrary.com]

Since 1990 (the World Bank income classification started in 1986), a total of 5101 out of 5323 (95.8%) editors were affiliated with HIC institutions, while 174 (3.2%), 43 (0.8%) and 1 (<0.1%) were affiliated with UMIC, LMIC and LIC institutions, respectively. There was a slight decrease in the proportion of editors affiliated with HIC institutions over the years from 242 out of 246 (98.4%) in 1990 to 1843 out of 1984 (92.9%) in 2020 (Fig. 5). This may be explained by the difference in data availability between 2020 (100% editorial boards available) and the preceding years (33–80% editorial boards available, Fig. 2).

Using information from journals, we were able to clarify the seniority of a total of 1219 editors on 2020 editorial boards with known gender and country of affiliation. Of these editors, 109 out of 259 (42%) women and 306 out of 960 men (32%) were senior editors. When considering geographical region, 287 out of 684 (42%) of editors from North America were senior, compared with 5 out of 16 (31%) from the Middle East and North Africa, 26 out of 87 (30%) from East Asia and Pacific, 86 out of 363 (24%) from Europe and Central Asia, 12 out of 63 (19%) from Latin America and the Caribbean and 0 out of 7 (0%) and 2 (<0.1%%) from South Asia and sub‐Saharan Africa, respectively. Considering the World Bank income classification, 397 out of 1115 (36%) editors affiliated with HIC institutions were senior, while 19 out of 93 (20%) editors affiliated with UMIC institutions were senior. Out of the 14 editors affiliated with LMIC institutions, none were senior.

**Figure 6:**
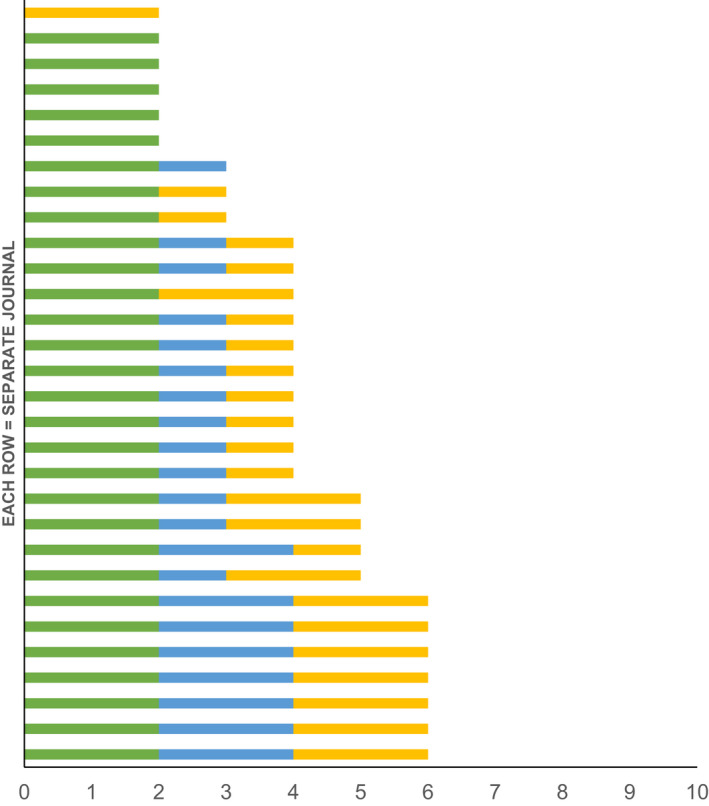
Individual scores of the composite editorial board diversity score of included anaesthesia journals in 2020 across the three domains: gender, country income level and geographic region. Absent bar = score 0 in that domain. Total score ≤ 5 = poor diversity; 6–7 = moderate diversity; ≥ 8 = good diversity [6]. Green, gender diversity (max 4); Blue, country income‐level diversity (max 3); yellow, geographic region diversity (max 3). [Colour figure can be viewed at wileyonlinelibrary.com]

The composite editorial board diversity scores for 2020 are shown in Fig. 6. The median (IQR [range]) composite editorial board diversity score was 4 (3–5 [2–6]) indicating poor to moderate diversity on the editorial boards. From 1990 to 2015, we were able to calculate a further 91 composite editorial board diversity scores (see online Supporting Information, Table S3), where the overall trend suggested improving composite editorial board diversity score over time. This was predominantly driven by an improvement in geographic region diversity.

## Discussion

To the best of our knowledge, this is the most comprehensive study looking at gender and diversity in the editorial boards of multiple journals in anaesthesia and the first to use the composite editorial board diversity score in this specialty. We found that women held a minority of editorial board positions in 2020, although there appears to have been an increase over time. Geographical representation outside HICs remains especially poor, with apparently much less progress than for gender representation. There is no apparent progress in the inclusion of editorial board members from LMIC since 1990, and there was not one single editorial board member identified from a LIC from 1990 to 2000. Our findings confirm previous similar studies assessing editorship in journals, which found similar under‐representation of women and people from low‐ and middle‐income countries in global health journals, psychiatry journals and the *Canadian Journal of Anesthesia* [5, 6, 17].

This study shows country income diversity on editorial boards is poor, with more than 90% of editors affiliated with HIC. If the target readership is physician anaesthetists, then evidence shows that, worldwide, 47.8% of physician anaesthetists are based in HIC, 40.2% in UMIC, 11.7% in LMIC and 0.002% in LIC [19]. The uneven distribution is due to a severe workforce crisis in LMIC and LIC [20]. However, if we believe that the research agenda should be driven by where patients and their needs are, then only 15.7% and 32.4% of the world's population are based in HIC and UMIC, respectively [21]. Multiple barriers exist to undertaking primary research in LMIC and LIC including: limited material; financial and human resources [22]; the requirement from most journals to write in English [23]; a feeling of editorial bias against their work [24]; and researchers with limited resources being unable to benefit from the citation advantage of open access publication due to high processing charges with limited or no waiver for LMIC and LIC [25].

We note that many journals included in this study are associated with national anaesthesia associations or societies. These require significant resources from their associated society to be successful and this may be a contributing factor to the dominance of editors from these high income countries. Some included journals may consider themselves to be primarily national, rather than international, journals. It could be argued that those associated with national associations may be expected to have editorial boards comprising editors who are mostly from that country. However, we argue that most journals included in this study now have a clearly defined international scope, with both international readership and authorship. More diverse editorial boards may contribute to journals being able to support and strengthen research capacity in low‐ and middle‐income countries.

Our findings show women are under‐represented on anaesthesia editorial boards. Available data from HICs show up to 47% of the anaesthesia workforce are women [19, 26, 27] although this may not compare with that of the overall global workforce. If this is a conservative target for journals to achieve, only five met or exceeded this in 2020. Reports demonstrate gender bias in academic medicine, and an important subset of this evidence focuses on journal‐level disparities [28]. Under‐representation of women in editorial boards prevents gender‐specific life experiences and perspectives being reflected in publications and impairs the output of female researchers [29]. Our findings suggest a plan of action should be formulated for journals to develop strategies for the recruitment, retention and advancement of women in anaesthesia authorship and journal editorial boards.

The composite editorial board diversity score shows at best moderate scores for the journals we assessed, and values were lowest for national income diversity. This score was initially developed to assess journals in global health [6] and as such it may have limited applicability to journals in anaesthesia. For example, it may not be as necessary for a major journal with a specific national target audience to have as regionally diverse an editorial board. A modification to the targets could be suggested but is beyond the scope of this study. The strength of this score, even in its current format, is the transparency, objectivity and consistency of comparison allowed between journals of the same and differing specialties. Data collection for this study included assessment of the roles given to members of the editorial boards. We identified 131 different titles used, with inconsistencies in the seniority of these and whether they were officially included in the editorial board [18], which impaired our assessment. The diversity of senior positions is relevant to assess more closely whether representation is tokenistic or genuine. It is therefore interesting to note, although without statistical measure, that the proportion of women in editorial boards receiving senior roles is greater than that of men even though the overall number in boards is lower. The same is not seen for country of affiliation.

Our study has several limitations. First, we were not able to obtain all historical editorial boards for analysis and use of 5‐yearly intervals may have inadequately represented their membership. Second, the gender determination of editorial board members was based on techniques used previously in similar studies but is at risk of bias: based on name rather than self‐assignment by the editors themselves and only in a binary format. We acknowledge that considering gender as binary is itself problematic, which was a methodological compromise to allow discussion of an under‐represented group (women). We cannot make any comment about representation of people who identify outside a gender binary. Third, some editors may be affiliated with an institution in a country different from that with which they would personally identify. This may have led to an underestimation of representation by geography and country income diversity. Finally, while not a limitation, we would like to acknowledge our study group composition, which is made up of six men and four women. Six authors are affiliated with HIC institutions, one with an UMIC institution and three with LMIC institutions, spread across five geographical regions. We have sought to bring the diversity of our experiences to this study.

Further research in this area should explore the representation of other components of diversity that may result in discrimination. Of note, ethnicity and its representation is a complex issue beyond the scope of our study. Further qualitative study could explore the impact of ethnicity, membership of other minority groups and living in a different country to that of birth, on representation and research priorities in academic anaesthesia.

We focused only on Science Citation Index Expanded‐listed journals; further research may seek to explore the diversity of anaesthesia journals listed elsewhere, including those listed in the Emerging Sources Citation Index and the Global Index Medicus. In order to enable equitable representation of women on editorial boards when compared with the anaesthesia workforce, additional research is needed to fully describe the current composition globally [27]. Further research in this area would also benefit from an intersectional approach, acknowledging that an individual has multiple, overlapping facets of their identity that can lead to marginalisation.

In conclusion, our findings show that the composition of editorial boards from Science Citation Index Expanded‐listed anaesthesia journals fails to represent the diversity of their readership and the populations they serve, with an over‐representation of male editors from HIC. A lack of diversity in editorial boards perpetuates barriers to publication, access to editorial advice and the development of academia in LMIC. It is also likely to contribute to the under‐prioritisation of knowledge from marginalised groups [4]. Conversely, diversity on the editorial boards can help to promote varied perspectives, be a step towards more equitable representation of the target audience and support submissions from researchers of diverse backgrounds.

This is a call to action. There are already some publishers committing to improving inclusion and diversity, promoting actions such as mentorship and inclusive shortlisting of applicants [30, 31]. These changes are long overdue. Journal publishers and editors have a responsibility to tackle the sources of bias that exist in their publications. We suggest that they address the composition of their editorial boards. In Box 1, we have compiled a set of recommendations, to help editorial boards improve the transparency around their board composition, enable editorial boards to use their key position in academia to support increased research capacity and assist reducing possible reviewer bias. We hope that they will use their power and privilege to promote a diverse editorial board, for the benefit of their contributors, their readership and the wider anaesthesia community.

Box 1 **Recommendations for actions by journals to improve transparency, increase research capacity and reduce bias against minoritised groups.**

**Table:** 

**Improving transparency**
• Collect and publish data annually concerning gender, geographical representation and other characteristics that may lead to discrimination within the editorial board, reviewers and authors [26]. • Develop, share and continually update policy statements relating to the diversity of gender, geographical representation and other characteristics on the editorial board and reviewers. These should include their intended targets and methods for improvement where there is inadequate representation [30]. • Require authors submitting manuscripts based on data from low‐ and middle‐income countries to provide a reflexivity statement based on table 1 from the consensus statement on equitable authorship from international partnerships [12].
**Increasing research capacity**
• Create editorial fellowships for applicants based outside HIC, with particular focus on candidates based in LMICs and LICs.* • Invite guest editors from under‐represented groups for special sections across the year [5]. • Develop a mentorship programme for experts based in low‐ and middle‐income countries to build up editorial skills. • Enable and encourage free pre‐submission review of papers for authors from low‐ and middle‐income countries, including support where publication language is a barrier [32]. • Enable free open access publication to authors from low‐ and middle‐income countries, and support use of this process by: ○ using non‐restrictive eligibility criteria. ○ offering to transfer submissions to open access if eligible authors have submitted through non‐open access pathways. ○ ensuring it is free rather than reduced cost [33].
**Reducing bias against minoritised groups** • Blind reviewers to the details of submitting authors (including gender and geographical affiliation).

*Anaesthesia is proceeding with this option.

## Supporting information


**Appendix S1.** Reflexivity statement.

**Table S1.** The number of editors of each gender on each editorial board in 2020, arranged according to the proportion of representation of women (from lowest to highest).
**Table S2.** Country affiliation of anaesthesia journal editors across all available data and in 2020, ranked according to the number of journal editors in 2020 (highest to lowest).
**Table S3.** The breakdown of composite editorial board diversity scores based on available data for the years 1990–2015 for different anaesthesia journals.

## Appendix 1 A

### Anaesthesia Journal Editorial Board Diversity and Representation Study Group (alphabetical order)

M. D. Bould, Children's Hospital of Eastern Ontario, University of Ottawa, Ottawa, Canada; R. Eng, Khmer‐Soviet Friendship Hospital, Phnom Penh, Cambodia; S. Glaze, S. Kudsk‐Iversen, S. McLure, Oxford University Hospitals NHS Trust, Oxford, UK; O. Higgins‐Gill, University of Guyana and Georgetown Public Hospital Corporation, Georgetown, Guyana; R. Inglis, Lao‐Oxford‐Mahosot Hospital, Wellcome Trust Research Unit, Laos PDR; S. Krouch, Sonja Kill Memorial Hospital, Krong Kampot, Cambodia; N. Shamambo, University Teaching Hospital, Lusaka, Zambia; V. Thwaites, St John's Hospital, Livingston, UK.
