# Supplementary material for: Trends in country and gender representation on editorial boards in anaesthesia journals: a pooled cross‐sectional analysis
Source: Anaesthesia. 2022 May 5;77(9):981–90. doi: 10.1111/anae.15733 (PMC9545632; doi:10.1111/anae.15733)
Supplement: Supplementary file 1 — Appendix S1. Reflexivity statement. [file ANAE-77-981-s001.docx]

# **Appendix S1 – Reflexivity Statement**

1. **How does this study address local research and policy priorities?**

This study addresses local research and policy priorities by quantifying representation by geography on editorial boards in journals in anaesthesia. The representation on editorial boards is likely to impact prioritisation of research, publications and their impact.

1. **How were local researchers involved in study design?**

The concept of the study was conceived by researchers in high-income countries (DB, SKI). A collaborative approach was taken and additional researchers from a diverse geography were invited to join the collaborative and design the study (RI, SK, RE, NS, OHG, VT, SJG, SM).

1. **How has funding been used to support the local research team?**

No funding has been used in the conduct of the study. Where academic resources were needed to obtain past issues of journals, a high-income institution’s library was used.

1. **How are research staff who conducted data collection acknowledged?**

All those who contributed to the study are listed as authors in a collaborative format.

1. **Do all members of the research partnership have access to study data?**

All members of the collaborative have access to data.

1. **How was data used to develop analytical skills within the partnership?**

Data collected did not undergo statistical analysis.

1. **How have research partners collaborated in interpreting study data?**

Manuscript preparation was shared between 3 members of the collaborative (OHG, SKI, SG) and all members then reviewed and provided feedback on the manuscript.

1. **How were research partners supported to develop writing skills?**

As above, manuscript preparation was a collaborative approach.

1. **How will research products be shared to address local needs?**

The study will be published as open access. Preliminary findings have been presented at a international conference which subsidised access to researchers from low and middle income countries. Once published, the paper will be shared with journals who were assessed in it.

1. **How is the leadership, contribution and ownership of this work by LMIC researchers recognised within the authorship?**

The study has a collaborative authorship to equalise recognition of authors from lower-middle, upper-middle-, and high-income countries. The study’s progression has been led by authors from high-income countries, but this has been in part due to their ability to contribute time to the study without funding and without impairing other research and clinical activities that are under-recognised.

1. **How have early career researchers across the partnership been included within the authorship team?**

The collaborative includes a range of research career experience. SG has led protocol and manuscript preparation with support from the more experienced SKI and RI.

1. **How has gender balance been addressed within the authorship?**

The authorship is six men (SKI, SG, SM, DB, RI, SK) and four women (RI, OHG, VT, NS).

1. **How has the project contributed to training of LMIC researchers?**

By highlighting the lack of representation, the study calls journals to action to recruit and train more researchers from low- and middle-income countries for their editorial boards.

1. **How has the project contributed to improvements in local infrastructure?**

This study has not contributed to improvements in infrastructure.

1. **What safeguarding procedures were used to protect local study participants and researchers?**

Data collection was from journals, there were no local study participants. Researchers did not require any specific safeguarding procedures.
