# Supplementary material for: Trends in country and gender representation on editorial boards in anaesthesia journals: a pooled cross‐sectional analysis
Source: Anaesthesia. 2022 May 5;77(9):981–90. doi: 10.1111/anae.15733 (PMC9545632; doi:10.1111/anae.15733)
Supplement: Supplementary file 2 — Table S1. The number of editors of each gender on each editorial board in 2020, arranged according to the proportion of representation of women (from lowest to highest). Table S2. Country affiliation of anaesthesia journal editors across all available data and in 2020, ranked according to the number of journal editors in 2020 (highest to lowest). Table S3. The breakdown of composite editorial board diversity scores based on available data for the years 1990–2015 for different anaesthesia journals. [file ANAE-77-981-s002.docx]

**Table S1** The number of editors of each gender (columns) on each editorial board (rows) in 2020, arranged according to proportion of representation of women (from lowest to highest).

| women (%) | Women (no.) | Men (no.) | not available | editors, total (No.) |
| --- | --- | --- | --- | --- |
| 0.0% | 0 | 19 | 0 | 19 |
| 2.5% | 2 | 75 | 4 | 81 |
| 3.7% | 2 | 52 | 0 | 54 |
| 8.7% | 2 | 21 | 0 | 23 |
| 10.9% | 6 | 49 | 0 | 55 |
| 12.5% | 4 | 28 | 0 | 32 |
| 14.2% | 17 | 103 | 0 | 120 |
| 15.3% | 9 | 50 | 0 | 59 |
| 16.1% | 27 | 139 | 2 | 168 |
| 17.0% | 9 | 44 | 0 | 53 |
| 17.6% | 6 | 28 | 0 | 34 |
| 18.1% | 15 | 67 | 1 | 83 |
| 19.0% | 4 | 17 | 0 | 21 |
| 19.1% | 9 | 37 | 1 | 47 |
| 19.5% | 8 | 33 | 0 | 41 |
| 19.9% | 34 | 137 | 0 | 171 |
| 21.7% | 5 | 18 | 0 | 23 |
| 22.2% | 8 | 28 | 0 | 36 |
| 22.4% | 34 | 118 | 0 | 152 |
| 23.3% | 7 | 23 | 0 | 30 |
| 24.2% | 16 | 50 | 0 | 66 |
| 25.0% | 13 | 39 | 0 | 52 |
| 26.0% | 19 | 54 | 0 | 73 |
| 26.7% | 12 | 33 | 0 | 45 |
| 26.8% | 45 | 123 | 0 | 168 |
| 27.3% | 6 | 16 | 0 | 22 |
| 30.0% | 9 | 20 | 1 | 30 |
| 33.3% | 30 | 59 | 1 | 90 |
| 35.7% | 10 | 18 | 0 | 28 |
| 38.0% | 41 | 66 | 1 | 108 |

**Table S2** Country affiliation of anaesthesia journal editors across all available data and in 2020, ranked according to the number of journal editors in 2020 (highest to lowest).

| Affiliated country | 2020 | All available data |
| --- | --- | --- |
| United States of America | 942 | 2552 |
| Germany | 176 | 297 |
| United Kingdom | 121 | 706 |
| Canada | 104 | 350 |
| Italy | 67 | 143 |
| Australia | 66 | 145 |
| Japan | 62 | 129 |
| Brazil | 61 | 68 |
| France | 60 | 211 |
| Belgium | 39 | 79 |
| Netherlands | 39 | 76 |
| Switzerland | 32 | 71 |
| China | 27 | 43 |
| Denmark | 24 | 51 |
| Israel | 14 | 37 |
| India | 12 | 21 |
| Spain | 12 | 31 |
| Sweden | 12 | 80 |
| Turkey | 12 | 14 |
| New Zealand | 9 | 19 |
| Norway | 9 | 21 |
| Austria | 8 | 21 |
| Singapore | 8 | 13 |
| Finland | 7 | 32 |
| Ireland | 7 | 17 |
| South Africa | 7 | 21 |
| Arab Republic of Egypt | 6 | 6 |
| Republic of Korea | 6 | 7 |
| Mexico | 4 | 11 |
| Portugal | 4 | 10 |
| Chile | 2 | 6 |
| Greece | 2 | 5 |
| Hungary | 2 | 7 |
| Iceland | 2 | 3 |
| Not available | 2 | 25 |
| Poland | 2 | 8 |
| Taiwan | 2 | 2 |
| Argentina | 1 | 3 |
| Honduras | 1 | 1 |
| Islamic Republic of Iran | 1 | 1 |
| Jordan | 1 | 1 |
| Lebanon | 1 | 2 |
| Qatar | 1 | 1 |
| Romania | 1 | 1 |
| Russian Federation | 1 | 4 |
| Saudi Arabia | 1 | 2 |
| Serbia | 1 | 1 |
| Thailand | 1 | 1 |
| United Arab Emirates | 1 | 1 |
| R. B. De Venezuela | 1 | 1 |
| Belarus | 0 | 1 |
| Bulgaria | 0 | 2 |
| Czech Republic | 0 | 5 |
| Indonesia | 0 | 1 |
| Uruguay | 0 | 2 |

**Table S3** The breakdown of composite editorial board diversity scores (CEBDS) based on available data for the years 1990-2015 for different anaesthesia journals.

| **Year** | **Gender diversity (max = 4)** | **Country income level diversity (max = 3)** | **Geographic region diversity (max = 3)** | **CEBDS (max = 10)** |
| --- | --- | --- | --- | --- |
| 1990 | 0 | 0 | 0 | 0 |
| 1995 | 2 | 0 | 0 | 2 |
| 2000 | 2 | 0 | 0 | 2 |
| 2005 | 0 | 0 | 0 | 0 |
| 2010 | 0 | 0 | 0 | 0 |
| 2015 | 0 | 1 | 1 | 2 |
| 1990 | 2 | 0 | 0 | 2 |
| 1995 | 2 | 0 | 0 | 2 |
| 2005 | 2 | 0 | 0 | 2 |
| 2010 | 0 | 0 | 1 | 1 |
| 2015 | 2 | 0 | 1 | 3 |
| 1990 | 2 | 0 | 1 | 3 |
| 1995 | 2 | 0 | 1 | 3 |
| 2000 | 2 | 0 | 1 | 3 |
| 2005 | 2 | 1 | 1 | 4 |
| 1990 | 2 | 0 | 0 | 2 |
| 1995 | 2 | 0 | 0 | 2 |
| 2000 | 2 | 0 | 1 | 3 |
| 2015 | 2 | 0 | 1 | 3 |
| 1990 | 2 | 0 | 0 | 2 |
| 1995 | 2 | 0 | 1 | 3 |
| 2000 | 2 | 0 | 1 | 3 |
| 2005 | 2 | 0 | 1 | 3 |
| 2010 | 2 | 0 | 1 | 3 |
| 2015 | 2 | 0 | 1 | 3 |
| 1990 | 0 | 0 | 0 | 0 |
| 1995 | 2 | 0 | 1 | 3 |
| 2000 | 2 | 0 | 1 | 3 |
| 2005 | 2 | 1 | 1 | 4 |
| 2015 | 2 | 0 | 1 | 3 |
| 1990 | 0 | 1 | 2 | 3 |
| 1995 | 2 | 0 | 1 | 3 |
| 2000 | 2 | 0 | 1 | 3 |
| 2005 | 2 | 0 | 1 | 3 |
| 2010 | 2 | 0 | 1 | 3 |
| 2015 | 2 | 0 | 1 | 3 |
| 1990 | 2 | 1 | 0 | 3 |
| 1995 | 2 | 1 | 0 | 3 |
| 2000 | 2 | 1 | 1 | 4 |
| 2005 | 2 | 1 | 1 | 4 |
| 2010 | 2 | 0 | 0 | 2 |
| 2015 | 2 | 0 | 0 | 2 |
| 2000 | 2 | 0 | 2 | 4 |
| 2005 | 2 | 0 | 2 | 4 |
| 2010 | 2 | 1 | 1 | 4 |
| 1990 | 2 | 1 | 2 | 5 |
| 1995 | 2 | 2 | 2 | 6 |
| 2000 | 2 | 1 | 2 | 5 |
| 2005 | 2 | 2 | 2 | 6 |
| 2010 | 2 | 1 | 2 | 5 |
| 2015 | 2 | 1 | 2 | 5 |
| 1990 | 2 | 0 | 1 | 3 |
| 1995 | 2 | 2 | 2 | 6 |
| 2000 | 2 | 0 | 0 | 2 |
| 2005 | 2 | 3 | 3 | 8 |
| 2010 | 2 | 2 | 3 | 7 |
| 2015 | 2 | 2 | 3 | 7 |
| 2000 | 2 | 0 | 2 | 4 |
| 2005 | 2 | 0 | 2 | 4 |
| 2000 | 2 | 0 | 2 | 4 |
| 2005 | 2 | 0 | 2 | 4 |
| 2015 | 2 | 2 | 2 | 6 |
| 1990 | 0 | 0 | 1 | 1 |
| 1995 | 2 | 0 | 1 | 3 |
| 2000 | 2 | 0 | 2 | 4 |
| 2005 | 2 | 0 | 2 | 4 |
| 2010 | 2 | 1 | 2 | 5 |
| 2000 | 0 | 0 | 0 | 0 |
| 2005 | 2 | 0 | 2 | 4 |
| 2010 | 2 | 0 | 1 | 3 |
| 2015 | 2 | 1 | 1 | 4 |
| 2000 | 2 | 1 | 3 | 6 |
| 2005 | 2 | 2 | 2 | 6 |
| 2010 | 2 | 2 | 3 | 7 |
| 2000 | 2 | 1 | 2 | 5 |
| 2005 | 2 | 2 | 2 | 6 |
| 2010 | 2 | 0 | 1 | 3 |
| 2015 | 2 | 0 | 1 | 3 |
| CEBDS ≤ 5 = poor diversity, CEBDS 6–7 = moderate diversity, CEBDS ≥ 8 = good diversity [6]. | | | | |
